# 31257 A Case Study of Needs Assessment Practices Using I-Corp Customer Discovery Protocols Alongside REDCap Surveys for CTSA Activities.

**DOI:** 10.1017/cts.2021.511

**Published:** 2021-03-30

**Authors:** Zahra Zunaed, Barbara Tafuto, Yasheca Ebanks, Peter Trinh, Doreen W. Lechner

**Affiliations:** 1 Ernest Mario School of Pharmacy, Rutgers, The State University of New Jersey, New Brunswick, NJ; 2 Department of Health Informatics, School of Health Professions, Rutgers, The State University of New Jersey, Newark, NJ; 3 New Jersey Alliance for Clinical and Translational Science, New Brunswick, NJ; 4 Rutgers Robert Wood Johnson Medical School, Rutgers, The State University of New Jersey, Piscataway, NJ

## Abstract

ABSTRACT IMPACT: The results from this study will improve needs assessment practices. OBJECTIVES/GOALS: The discovery phase in project development is necessary to better understand the needs and requirements of the intended market. This paper compares the outcomes of two virtual data collection methodologies, NSF I-Corps Customer Discovery interviews and REDCap surveys, for a needs assessment. METHODS/STUDY POPULATION: Clinical and Translational Science Award (CTSA) Directors and Academic Administrators across the Consortium were asked about the types of skills needed to assess clinical research professional competencies and the need for a competency-based self-assessment tool (CBST). Parallel methods were used to extract qualitative and quantitative data. The first approach was to conduct interviews using I-Corps customer discovery guidelines, and data was collected using Innovation Within software. Targeted requests were sent via cold email outreach to 102 individuals within 63 CTSA hubs. The second approach involved the use of the NJ ACTS Training and Education Offering Inventory REDCap Survey which was distributed via LISTSERV to 63 CTSA hubs. Response rates and user insights from each method were compared. RESULTS/ANTICIPATED RESULTS: Twenty-one of 63 CTSA hubs responded to the survey (response rate: 33%) while 18 of 63 hubs participated in an interview (response rate: 28%). Twenty-two individuals out of 102 were interviewed (response rate: 21%). Fifty-nine percent of interviewees and 62% of survey respondents indicated a clear need for a CBST; types of responses varied. Forty user insights were obtained from ten interviews. Two insights were gained in the survey from the eight who were prompted to fill out the free-text response. Both survey participants and interviewees indicated that communication and team science soft skills were the most important competencies. Regarding hard skills, interviewees preferred written skills while survey participants favored ‘scientific design and concept’ skills. DISCUSSION/SIGNIFICANCE OF FINDINGS: Results suggest the use of a survey or an interview for a needs assessment is dependent on several factors: need for insights, burden of time, desire to obtain quantitative vs. qualitative data, and question format. The interview was more effective than the survey in addressing the key question and obtaining insights from the intended market.

